# Comprehensive analysis of the role of a four-gene signature based on EMT in the prognosis, immunity, and treatment of lung squamous cell carcinoma

**DOI:** 10.18632/aging.204878

**Published:** 2023-07-17

**Authors:** Feng Li, Hui Wang, Can Wang, Yun Li, Jing-Yan Song, Ke-Yi Fan, Chao Li, Quan-Lin Ma, Qi Yu, Shuang-Ping Zhang

**Affiliations:** 1 Department of Cell Biology, Shanxi Province Cancer Hospital, Chinese Academy of Medical Sciences, Cancer Hospital Affiliated to Shanxi Medical University, Taiyuan, China; 2 Department of Thoracic Surgery, Yangquan First People's Hospital, Yangquan, China; 3 Shanxi Medical University, School of Management, Taiyuan, China; 4 The First Clinical Medical College, Shanxi Medical University, Taiyuan, China; 5 Department of Thoracic Surgery, Shanxi Province Cancer Hospital, Shanxi Hospital Affiliated to Cancer Hospital, Chinese Academy of Medical Sciences, Affiliated Tumor Hospital of Shanxi Medical University, Taiyuan, China; 6 Department of Cardiothoracic Surgery, Shanxi Fenyang Hospital, Fenyang, China; 7 Institute of Medical Data Science, Shanxi Medical University, Taiyuan, China

**Keywords:** lung squamous cell carcinoma, epithelial-mesenchymal transition, immunity, treatment

## Abstract

Epithelial-mesenchymal transition (EMT), a biological process through which epithelial cells transform into mesenchymal cells, contributes to tumor progression and metastasis. However, a comprehensive analysis of the role of EMT-related genes in Lung squamous cell carcinoma (LUSC) is still lacking. In this study, data were downloaded from available databases, including The Cancer Genome Atlas (TCGA) database and the Gene Expression Omnibus (GEO) database. The association between differentially expressed EMT-related genes (EMT-RDGs) and LUSC prognosis, drug sensitivity, mutation, and immunity was analyzed using bioinformatics methods. In the results, Lasso and univariate Cox regression analyses identified four EMT-RDGs that were differentially expressed, and used to establish a prognostic model capable of distinguishing between high- and low-risk groups. Then, prognostic factors were identified by multivariate Cox regression analysis and used to construct a nomogram. The high-risk group had a significantly poorer prognosis than the low-risk group. The tumor immune environment was significantly different between the two groups, with the low-risk group exhibiting a better response to immunotherapy. In addition, the half-maximal inhibitory concentration prediction indicating that the constructed model could effectively predict sensitivity to chemotherapy. This study provides new reference for further exploration of new clinical therapeutic strategies for LUSC.

## INTRODUCTION

Lung cancer is the most commonly diagnosed cancer worldwide (11.6%) and is the leading cause of cancer-related deaths (18.4%) [1]. Lung squamous cell carcinoma (LUSC) is a prevalent form of non-small cell lung cancer (NSCLC), accounting for approximately 30% of all lung cancers [2]. Despite advances in chemotherapy and molecular-targeted therapies in recent years, the prognosis for LUSC patients remains very poor [3, 4].

Metastasis is the most prominent feature of cancer cells and the primary cause of death in 90% of cancer patients. The epithelial-to-mesenchymal transition (EMT) is originally known as a process during embryonic development in which cells acquire mesenchymal phenotype and lose epithelial phenotype. Persistent EMT is essential for dissemination from primary tumors, with the progress of EMT, the tumor cells obtain motile and invasive phenotype, the loss of cell-cell adhesion capacity and increased motility and invasion ability during EMT, which results in tumor cells escaping from primary tumors and invading the bloodstream or lymphatic system [5]. According to the transient EMT model, a subsequent mesenchymal - epithelial transition (MET) step can play a role in tumor metastasis [6]. Additionally, EMT can promote tumor cell proliferation, inhibit apoptosis, reduce cellular senescence, and promote immunosuppression [7]. EMT activation is the primary mechanism in the generation of cancer stem cell (CSC) [8], and it is regulated by a series of EMT-activated transcription factors (EMT-TFs), including the SNAIL1, TWIST, and ZEB families.

An extensive study has been conducted on the relationships between EMT and prognosis in non-small cell lung cancer in recent years. Schliekelman et al. [9] discovered a correlation between EMT phenotype and NSCLC cell invasion ability. Byres et al. [10] developed a 76-gene EMT signature to investigate the clinical responses to inhibitors in NSCLC patients. EMT-related genes (ERGs) have significant clinical relevance in NSCLC. However, there are no systematic studies of ERGs and their relationships with LUSC prognosis and treatment efficacy. As such, we used TCGA data and GEO data as the training and validation datasets, respectively. The two sets were used to screen differentially expressed EMT-related genes (EMT-RDGs) and construct a prognostic model. Then, we investigated their relationship with prognosis, immune infiltration, drug sensitivity, and gene mutation in LUSC patients, which can provide a basis for clinical treatment of LUSC patients.

## MATERIALS AND METHODS

### Data collection and collation

Gene expression data (FPKM value) and clinical information on LUSC were down-loaded from the TCGA database(https://gdc.xenahubs.net). FPKM were then transformed to TPM. The validation dataset (GSE73403) with prognostic information was downloaded from the GEO database (https://www.ncbi.nlm.nih.gov/geo/, TPM, transcripts per million).

The gene mutations and gene copy number variants information of 492 LUSC from the TCGA database is publicly available via the GDC Data Portal (https://portal.gdc.cancer.gov/). mRNAsi indexes for LUSC cases in TCGA were obtained from previous studies.

The data on Cancer-associated transcription factors (TFs) used in subsequent studies were downloaded from the Cistrome Cancer database.

### Selection of EMT-related genes

The ERGs list was obtained from the EMT gene database (http://dbemt.bioinfo-minzhao.org). Other ERGs were obtained from Molecular Signatures Database (MsigDB) (http://www.broad.mit.edu/gsea/msigdb/), specifically Hallmarkdata set (h.all.v7.2.symbols.gmt), GO data set (c5.bp.v7.2.symbols.gmt), KEGG gene set (c2.cp.kegg.v7.2.symbols.gmt), BioCarta gene set (c2.cp.biocarta.v7.2.symbols.gmt), PID gene set (c2.cp.pid.v7.2.symbols.gmt), and Reactome gene set (c2.cp.reactome.v7.2.symbols.gmt). These genes were summarized for inclusion in this study.

### Identification of EMT-RDGs

*P*-value < 0.05 and |logFC| > 0.32 were set as inclusion criteria for selection of differentially expressed genes (DEGs) between tumor and normal samples using the limma R package [11]. The EMT-RDGs were obtained by intersecting previously obtained differential genes with the ERGs. To elucidate the potential biological function of EMT-RDGs, the GO enrichment analyze, including the terms “biological process (BP),” “cellular component (CC),” and “molecular function (MF)” and KEGG pathway enrichment analysis were implemented using the ClusterProfiler (https://www.bioconductor.org/packages/release/bioc/html/clusterProfiler.html) package [12–15]. The pvalue < 0.05 was regarded as the screening criteria, and the dot plot function was used to visualize the results. Gene Set Variation Analysis (GSVA) was performed to generate the composite score of each gene set and to analyze the potential biological function alterations of different samples.

### Establishment of an ERGs-prognostic model based on Cox regression and lasso regression analysis

Samples with a shorter than 90-day survival time were excluded. Univariate Cox regression analysis was performed to identify differentially expressed genes associated with survival. Least absolute shrinkage and selection operator (LASSO) regression analysis was performed to select ERGS expression features for prognostic model-building for LUSC patients by R package ‘glmnet’. Feature coefficients were plotted against shrinkage parameter (Lambda) after performing linear regression between ERGS expression using LASSO in the training cohort (TCGA dataset). The minimum Lambda which resulted in the least error was identified after cross-validation of regression between weighted expression level of 4 genes: Snail family transcriptional repressor 1 (SNAI1), Mothers against decapentaplegic homolog 7 (SMAD7), Bone morphogenetic protein 2 (BMP-2), and Regulator of G-protein signalling 3 (RGS3). An EMT-RDGs signature was ultimately established to predict prognosis. We calculated risk scores equal to the sum of the products of gene expression levels and the corresponding coefficients (∑expression levels × coefficients) as follows:

(Risk score = SNAI1 expression × 0.007 + SMAD7 expression × 0.005 + BMP2 expression × 0.013 + RGS3 expression × 0.065)

### Construction of a transcription factors co-expression network

To evaluate the regulatory effect exerted by TFs on EMT-RDGs, we also examined their correlation. Pearson correlation analysis was used to perform correlation analysis. The 795 TFs were obtained from the database, and the TFs with empty values were eliminated. Correlation coefficient > 0.5 and FDR < 0.001 were set as the cutoff values for selection to analyze the relationship between 530 TFs and EMT-RDGs.

### Validation of the performance of prognostic models

The training cohort was divided into low-risk and high-risk groups using the median risk score as the cutoff point. Principal component analysis (PCA) and t-SNE were used to assess the grouped samples and expression patterns. Survival analysis was performed using the R package survminer to determine the survival difference be-tween the two groups. We then generated ROC curves to evaluate the performance of the prognostic model. Univariate and multivariate Cox regression analyses were performed to determine whether the four-gene model was an independent prognostic factor for LUSC. A nomogram was constructed based on age, gender, stage, smoking status, and risk score. In addition, calibration curves were plotted to assess the consistency between actual and predicted survival rates. The four-gene model was validated in an independent patient cohort (GSE73403).

### Assessment of immune infiltration and analysis of immune checkpoints

CIBERSORT was used to estimate the proportions of 22 sorted immune cell subtypes between the high- and low-risk LUSC patients. We employed the ESTIMATE algorithm to determine the immune and stromal scores, which reflect the enrichment of immune and stromal cell gene signatures, respectively. Tumor Immune Dysfunction and Exclusion (TIDE) was performed to investigate immune response. The ggpubr package was used to draw boxplots displaying comparisons of cytolytic activity scores, T cell inflammation scores, and mRNAsi indexes across different subgroups.

### Evaluation of drug susceptibility

We used the R package pRRophetic to predict the half-maximal inhibitory concentration (IC50) of chemotherapy drugs in the high- and low-risk groups of LUSC patients, and examined the sensitivity of different patients to chemotherapy drugs. In addition, the ridge regression model was constructed by integrating the gene expression profiles of cell lines from Genomics of Cancer Drug Sensitivity (GDSC, https://www.cancerrxgene.org/) and the TCGA data portal. Model accuracy was evaluated using a10-fold cross-validation.

### Gene mutation analysis

The gene mutation data were obtained from the publicly available TCGA database via the GDC Data Portal (https://portal.gdc.cancer.gov/) using “MuTect2 Variant Aggregation and Masking”. Then, we used the malftools package in R [16] to analyze and visualize the SNP difference between the high- and low-risk groups. Significantly mutated genes (SMGs) were identified using MuSigCV (mutation significance with covariates). Agene was considered SMG if it satisfied the condition for statistical significance (*q* < 0.05) at MuSigCV.

### Statistical analysis

All statistics and visualization were performed using the R software 4.1.1. All results were considered statistically significant when *P* <  0.05, ^*^P < 0.05, ^**^P < 0.01, ^***^P < 0.001 and ^****^P < 0.0001 denoted statistical significance.

### Cell culture and qRT-PCR

A total of 10 pairs of surgically resected cancer tissues and adjacent non-tumorous tissues were collected from patients with a pathological diagnosis of LUSC at the Shanxi Provincial Cancer Hospital between January 2021 and January 2022. The mRNA ex-pression levels of selected EMT-RDGs were validated using qRT-PCR. Total cellular RNA was extracted using Trizol reagent (Invitrogen, CA, USA). Reverse transcription of RNA to cDNA was performed using PrimeScript™ RT Master Mix (Perfect Real Time) (Takara RR036A). Quantitative real-time PCR was performed using the GoTaq^®^ qPCR Master Mix kit (Promega A6001) to determine the mRNA expression level of the hub genes. The expression levels of target genes were determined by qRT-PCR performed in triplicate on a Vii7 Q-PCR System (ABI, USA). Melting curves were generated at the end of amplification to confirm the specificity of the PCR product. Table 1 depicts the synthesis of the primers used in this study. To determine the relative expression of each target gene, GAPDH was used as the reference gene. Relative quantification was calculated using the comparative 2^−ΔΔCt^ method.

**Table 1:** Primer sequences used for qRT-PCR.

**Gene Name**		**Sequence**
SNAI1	FORWARD	GCCTAGCGAGTGGTTCTTCTG
SNAI1	REVERSE	TAGGGCTGCTGGAAGGTAAA
SMAD7	FORWARD	ATGTTCAGGACCAAACGATCT
SMAD7	REVERSE	GGATGGTGGTGACCTTTGG
BMP2	FORWARD	GACGTTGGTCAACTCTGTTAAC
BMP2	REVERSE	GTCAAGGTACAGCATCGAGATA
RGS3	FORWARD	CAGTGAGATCATCCTACTCGTG
RGS3	REVERSE	CAGTTCTTCTCCCGTTTGTTG
PMEPA1	FORWARD	CGTAGGTGAAAAGGCAGAACA
PMEPA1	REVERSE	GACACAGCTCAACAAAGAAACGT
LOXL2	FORWARD	ACAGAATGTGAAGGAGACATCC
LOXL2	REVERSE	TGATGTTGTTGGAGTAATCGGA
PLOD2	FORWARD	GGATGCAGATGTTGTTTTGACA
PLOD2	REVERSE	GCTTTCCATGACGAGTTACAAG
MMP14	FORWARD	CAAGATTGATGCTGCTCTCTTC
MMP14	REVERSE	ACTTTGATGTTCTTGGGGTACT
SPOCK1	FORWARD	CAGAAACTGGAATCCCAACAAG
SPOCK1	REVERSE	TTGCACTTGACCAAATTCGAAG
DCN	FORWARD	GACAACAACAAGCTTACCAGAG
DCN	REVERSE	TGAAAAGACTCACACCCGAATA
GAPDH	FORWARD	TGACTTCAACAGCGACACCCA
GAPDH	REVERSE	CACCCTGTTGCTGTAGCCAAA

### Data availability

The datasets analyzed for this study can be found in the online repositories. The data underlying this study are freely available from TCGA database (https://gdc.xenahubs.net), the GSE73403 dataset (https://www.ncbi.nlm.nih.gov/geo/), GDC Data Portal (https://portal.gdc.cancer.gov/) and the Cistrome Cancer database.

## RESULTS

### Data collection

**Figure 1:**
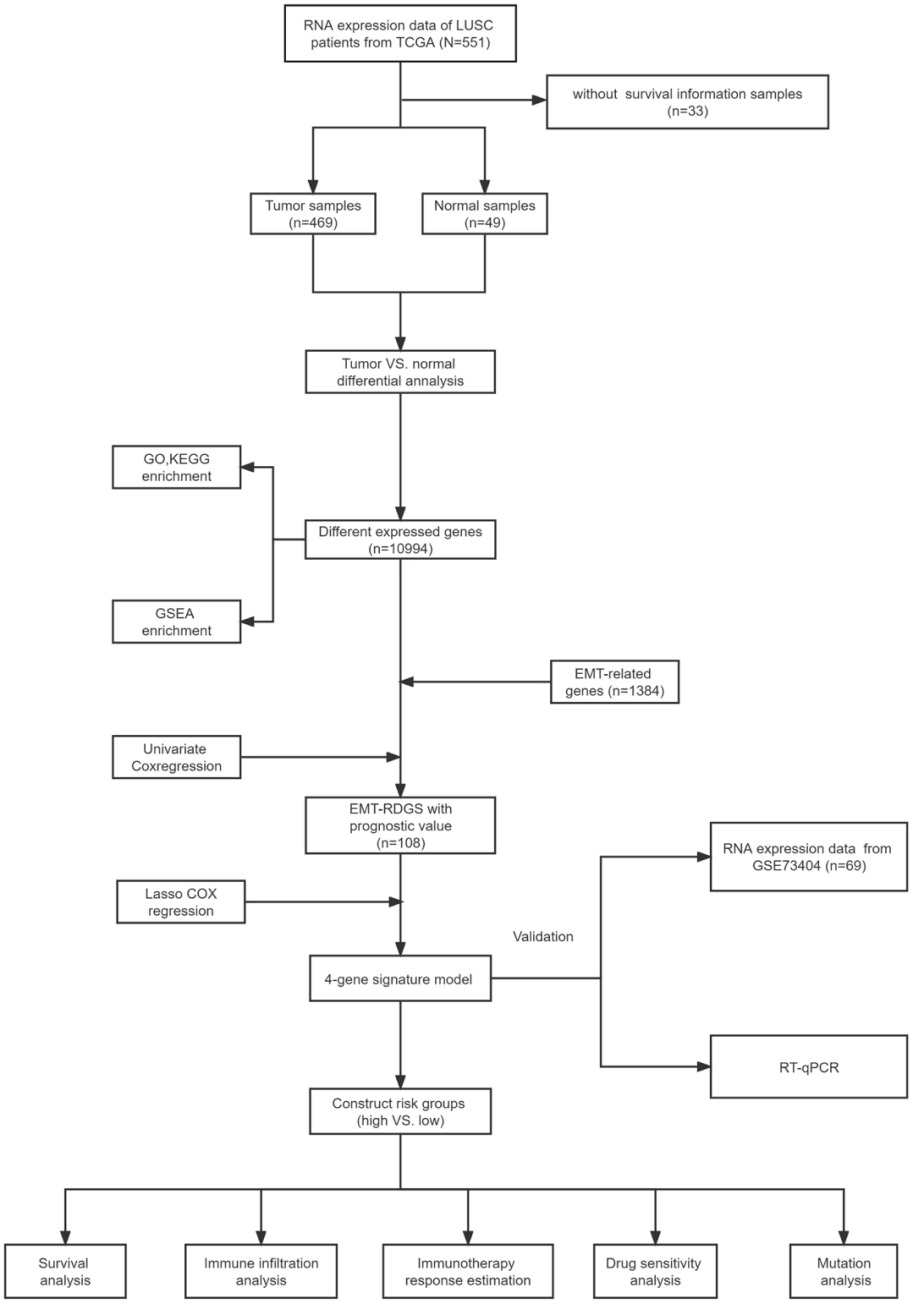
The flow chart of our study.

The flow chart of our study is shown in Figure 1. After removing outliers (Pearson’s correlation coefficients < 0.8), a total of 469 tumor samples and 49 normal samples from TCGA remained in the training set, and 69 patients from the validation dataset (GSE73403) containing prognostic information were included as the testing set. Table 2 provides a summary of the clinical information of LUSC patients.

**Table 2:** The clinical information of LUSC patients from TCGA and GEO databases.

	**TCGA *N* = 469**	**GEO *N* = 69**
Age (%)		
≤65	181 (38.6)	47 (68.1)
>65	283 (60.3)	22 (31.9)
Unknown	5 (1.1)	–
Gender (%)		
Female	119 (25.4)	4 (5.8)
Male	350 (74.6)	65 (94.2)
T (%)		
T1	108 (23.0)	4 (5.8)
T2	273 (58.2)	42 (60.9)
T3	67 (14.3)	20 (29.0)
T4	21 (4.5)	3 (4.3)
*N* (%)		
N0	300 (63.9)	35 (50.8)
N1	122 (26.0)	17 (24.6)
N2	37 (7.9)	17 (24.6)
N3	5 (1.1)	–
NX	5 (1.1)	–
M (%)		
M0	388 (82.7)	69 (100.0)
M1	6 (1.3)	–
MX	75 (16.0)	–
Stage (%)		
I	228 (48.6)	25 (36.2)
II	156 (33.3)	21 (30.4)
III	75 (16.0)	23 (33.3)
IV	6 (1.3)	–
Unknown	4 (0.8)	–
Smoking (%)		
Yes	440 (93.8)	58 (84.1)
No	17 (3.6)	11 (15.9)
Unknown	12 (2.6)	–
Survival status		
Alive	266 (56.7)	41 (59.4)
Dead	203 (43.3)	28 (40.6)
OS days (Median; Quartile)	973.5 [669.0, 1124.2]	943.7 [883.3, 1268]

### Screening of EMT-RDGs

**Figure 2:**
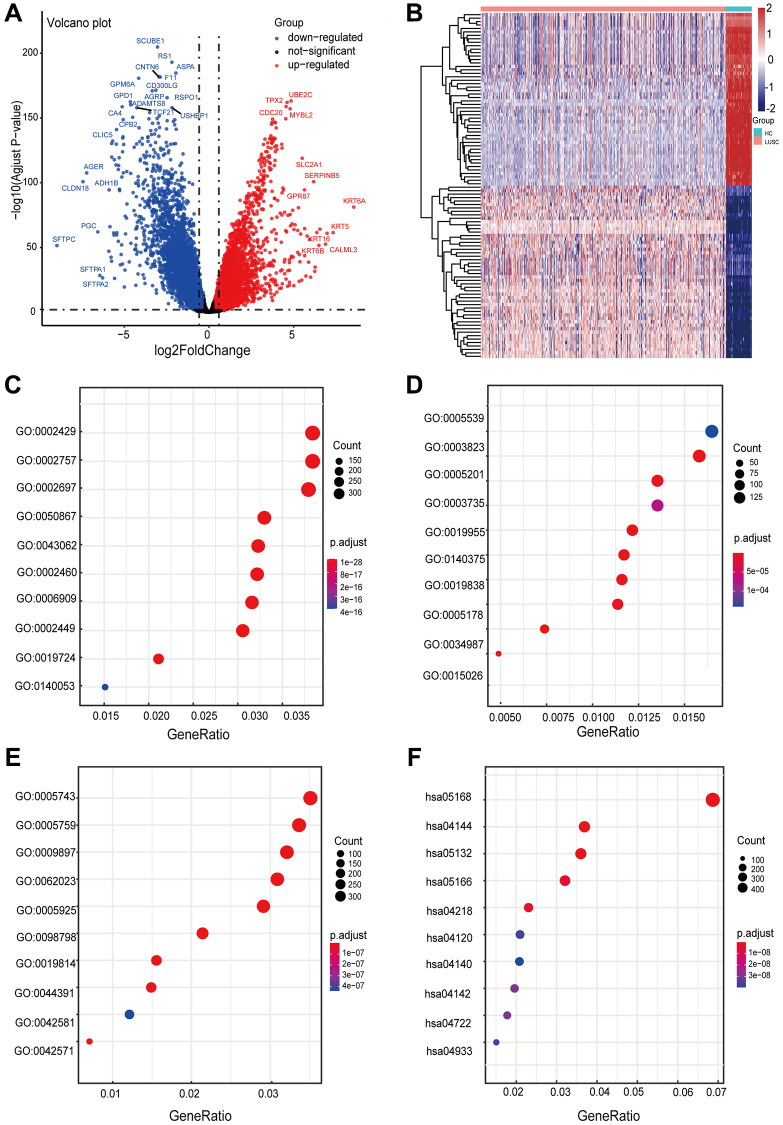
**Visualization of differentially expressed EMT-related genes and functional enrichment analysis of DEGs in LUSC.** (**A**) The volcano plot for differentially expressed genes between LUSC and normal samples. (**B**) The heatmap for differentially expressed genes between LUSC and normal samples. (**C**–**F**) Significantly enriched pathways in biological processes (BP), molecular function (MF), cellular components (CC) and KEGG pathway. The size of the dots represents the number of enriched genes, while their color indicates the degree of enrichment.

There were 10,994 DEGs between LUSC samples and normal samples, including 4968 upregulated genes and 6026 downregulated genes (Figure 2A). The heatmap and volcano plot were performed to visualize differentially expressed genes (Figure 2B). We analyzed DEGs using GO and KEGG analyses to further investigate the biological functions and signaling pathways involved in the occurrence and progression of diseases (Supplementary Figure 1A and 1B). GO enrichment results revealed that several terms were enriched for biological process (BP) (Figure 2C), molecular function (MF) (Figure 2D), and cellular component (CC) (Figure 2E). For BP, the DEGs were significantly enriched in the ‘immune response-activating cell surface receptor signaling pathway’, ‘immune response-activating signal transduction’, and ‘regulation of immune effector process’. For MF, the DEGs were enriched in ‘glycosaminoglycan binding’, ‘antigen binding’, and ‘extracellular matrix structural constituent’. For CC, the DEGs were enriched in ‘mitochondrial inner membrane’, ‘mitochondrial matrix’, and ‘external side of plasma membrane’. KEGG enrichment analysis revealed that these genes were primarily related to ‘herpes simplex virus 1 infection’, ‘endocytosis’, and ‘salmonella infection’ (Figure 2F). The biological functions were associated with the extensive fusion of human alveolar epithelial cells [17], the dissemination and colonization of metastatic cells [18], cell apoptosis, and the development of lung cancer [19]. Gene set enrichment analysis based on MsigDB revealed that differential genes were particularly enriched along multiple pathways, including TRANSFERASE_ACTIVITY_TRANSFERRING_ONE_CARBON_GROUPS and CELL_CYCLE, etc, indicating a close relationship between differential genes and EMT. Thus, 883 EMT-RDGs were selected by intersecting DEGs with 1384 ERGs, including 464 upregulated and 419 downregulated genes.

### Establishment of the four-gene prognostic model

The candidate 883 EMT-RDGs were subjected to univariate Cox regression analysis, and we obtained 108 differentially expressed genes related to survival. Then, 108 genes were filtered using LASSO regression analysis (Supplementary Figure 2A and 2B). Four EMT-RDGs, *SNAI1*, *SMAD7*, *BMP2*, and *RGS3* were eventually associated with the prognosis of LUSC. The four EMT-RDGs were used to construct the prognostic model.

### Construction of a transcription factors co-expression network

Correlation analysis between TFs and EMT-RDGs revealed that abnormal expression of EMT-RDGs was significantly associated with 29 TFs. Therefore, to better explain this association, we constructed a TFs-based Sankey diagram. There are 29 TFs and 4 EMT-RDGs in the Sankey diagram (Supplementary Figure 3).

### Performance validation of the predictive models

**Figure 3:**
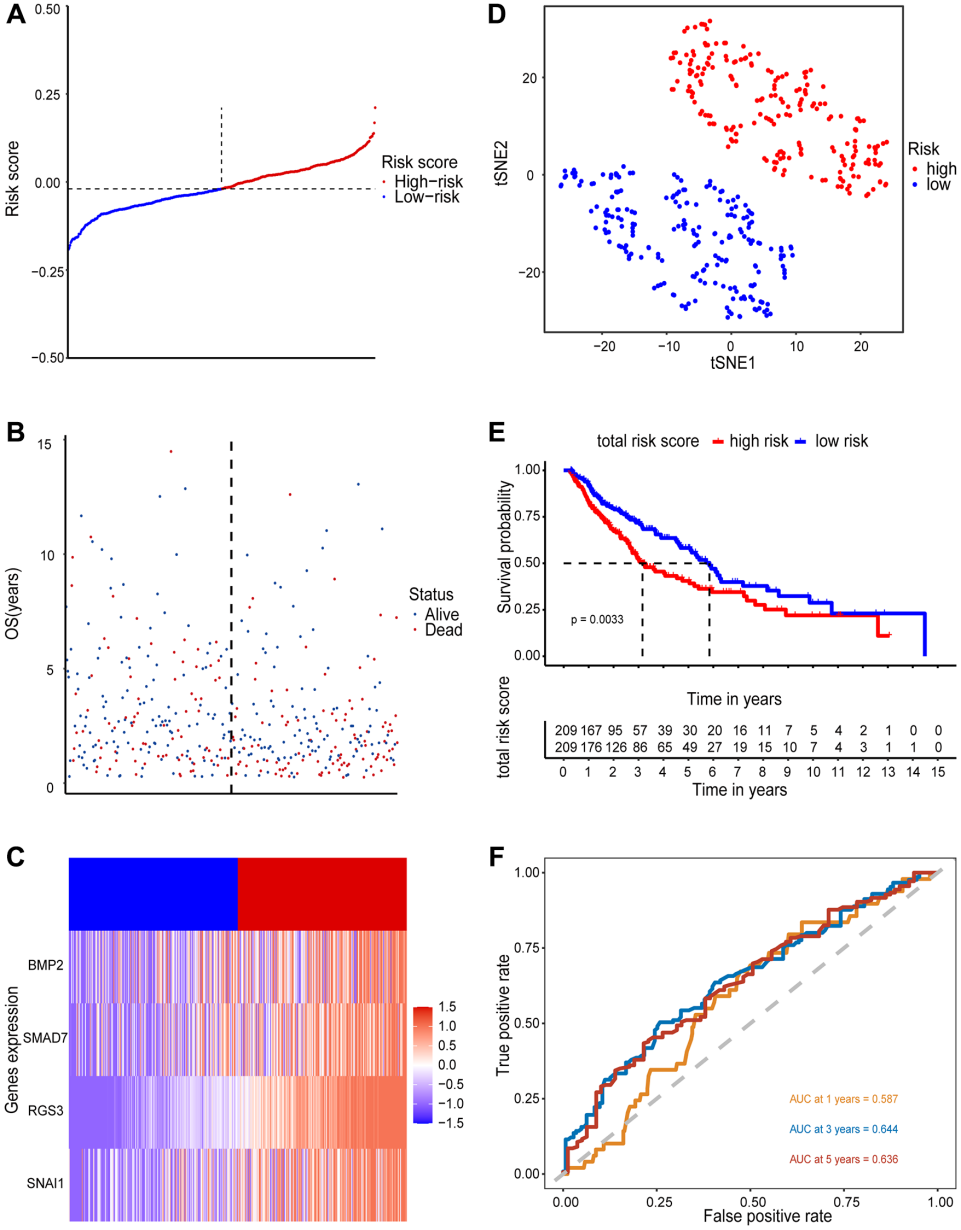
**Prognostic value of 4 EMT-RDGs in the training set.** (**A**) A risk curve based on the risk score of each sample. (**B**) The scatter plot is based on the survival status of each sample, the blue and red dots represent survival and death, respectively. (**C**) A heatmap of 4 EMT-RDGs. (**D**) t-SNE plot of high-and low-risk groups based on the four-gene prognostic model. (**E**) Kaplan-Meier curve for training set overall survival. (**F**) ROC curves for the 1, 3, and 5-year survival prediction.

The 418 tumor samples were acquired after filtering samples who had survival times of less than 3 months. Based on the risk score formula and the calculated median risk score, LUSC patients were divided into high-risk (*n* = 209) and low-risk groups (*n* = 209). The clinicopathological characteristics of the two groups are shown in Supplementary Figure 4. The risk score and the corresponding survival status of LUSC patients were illustrated by the risk curve and scatter plots (Figure 3A and 3B), and the expression-identifications of 4 EMT-RDGs between the high- and low-risk groups were compared by the heatmap (Figure 3C). To determine if our prognostic model could identify LUSC patients, we used PCA analysis and t-SNE to examine the distribution patterns of the high- and low-risk groups (Figure 3D).

According to our four-gene model, the high- and low-risk groups were reasonably distinct. Similar findings were obtained when the four-gene model was also applied to the validation set (Supplementary Figure 5A–5D). The Kaplan-Meier survival analysis revealed that the high-risk group had a poorer overall survival (OS) than the low-risk group (*P* < 0.001) in both the training and validation sets (Figure 3E and Supplementary Figure 5E). The ROC curves (Figure 3F) were plotted to assess the prognostic performance of the model. The area under the curve (AUC) for 1-, 3- and 5- year OS predictions were 0.587, 0.644, and 0.636, respectively. In the validation sets, the AUC for 1-, 3- and 5- year OS predictions were 0.789, 0.695, and 0.846, respectively (Supplementary Figure 5F). The above findings indicated that the four-gene model could accurately predict the prognosis of LUSC patients.

### The four-gene model as an independent prognostic factor in LUSC patients

**Figure 4:**
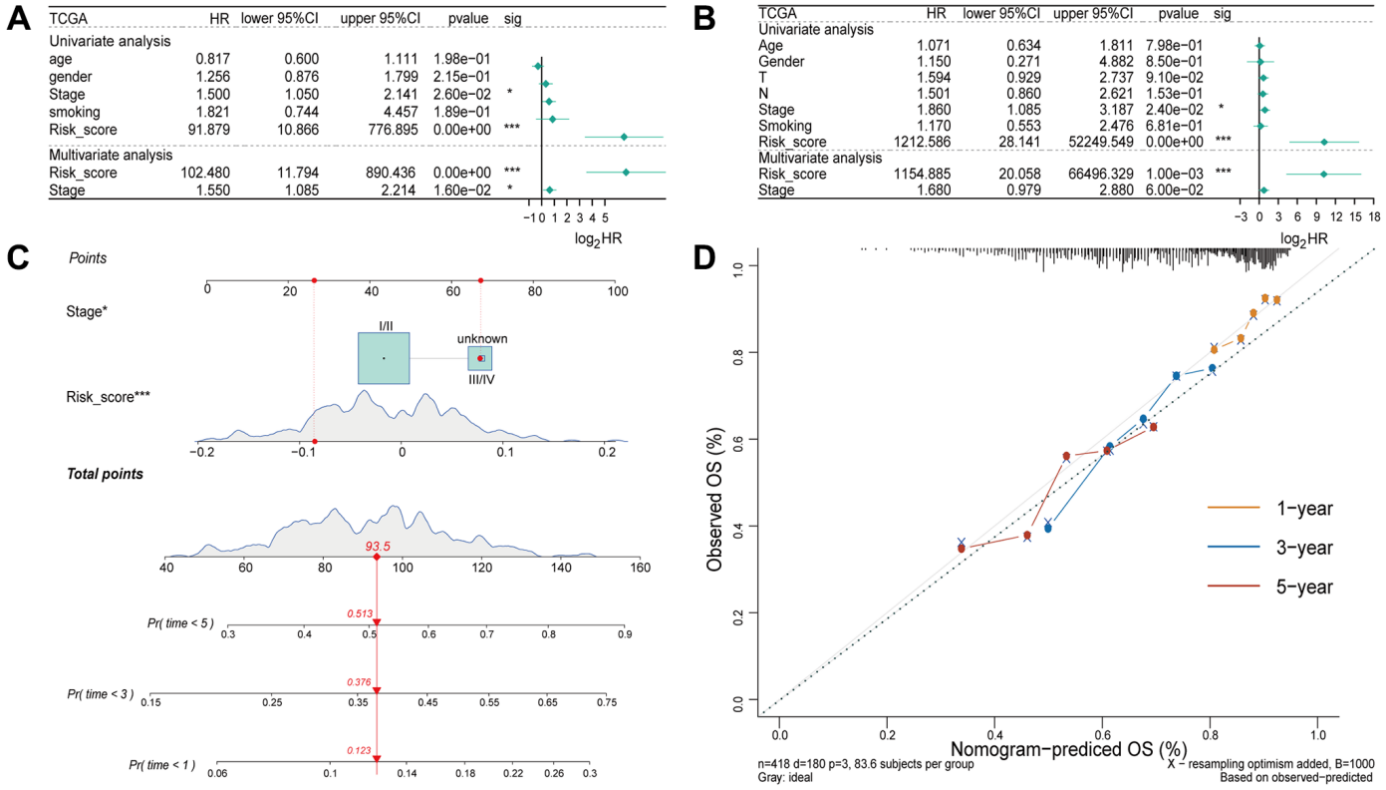
**Independent prognostic analysis of the four-gene model.** (**A**) Univariate and multivariate Cox regression analysis of the risk score and clinicopathological characteristics in the training set. (**B**) Univariate and Multivariate Cox regression analysis of the risk score and clinicopathological characteristics in the validation set. (**C**) A nomogram for prognostic prediction based on risk score and other clinicopathological factors in patients with LUSC. (**D**) Calibration curve for evaluating the predictive accuracy of the prognostic model.

Univariate and multivariate Cox regression analyses were performed to determine if the risk score and clinicopathological characteristics (age, gender, stage, and smoking status) could be used as independent risk factors of overall survival. Univariate and multivariate Cox regression analysis demonstrated that the stage and risk score was significantly associated with OS (Figure 4A). In the validation set, both univariate and multivariate Cox regression analysis revealed a significant association between the risk score and OS (Figure 4B). The aforementioned findings indicated that the four-gene model had a high clinical utility.

Based on the four-gene model and clinicopathological characteristics, a nomogram was constructed to predict the survival rate of individuals based on EMT-RDGs and clinical factors (Figure 4C). Additionally, a calibration curve was constructed to evaluate the predictive accuracy of the prognostic model (Figure 4D). The value of the C index was 0.62. This indicates that the prognostic model can be used to predict prognosis of LUSC patients.

### EMT-RDGs-based immune infiltration analysis

**Figure 5:**
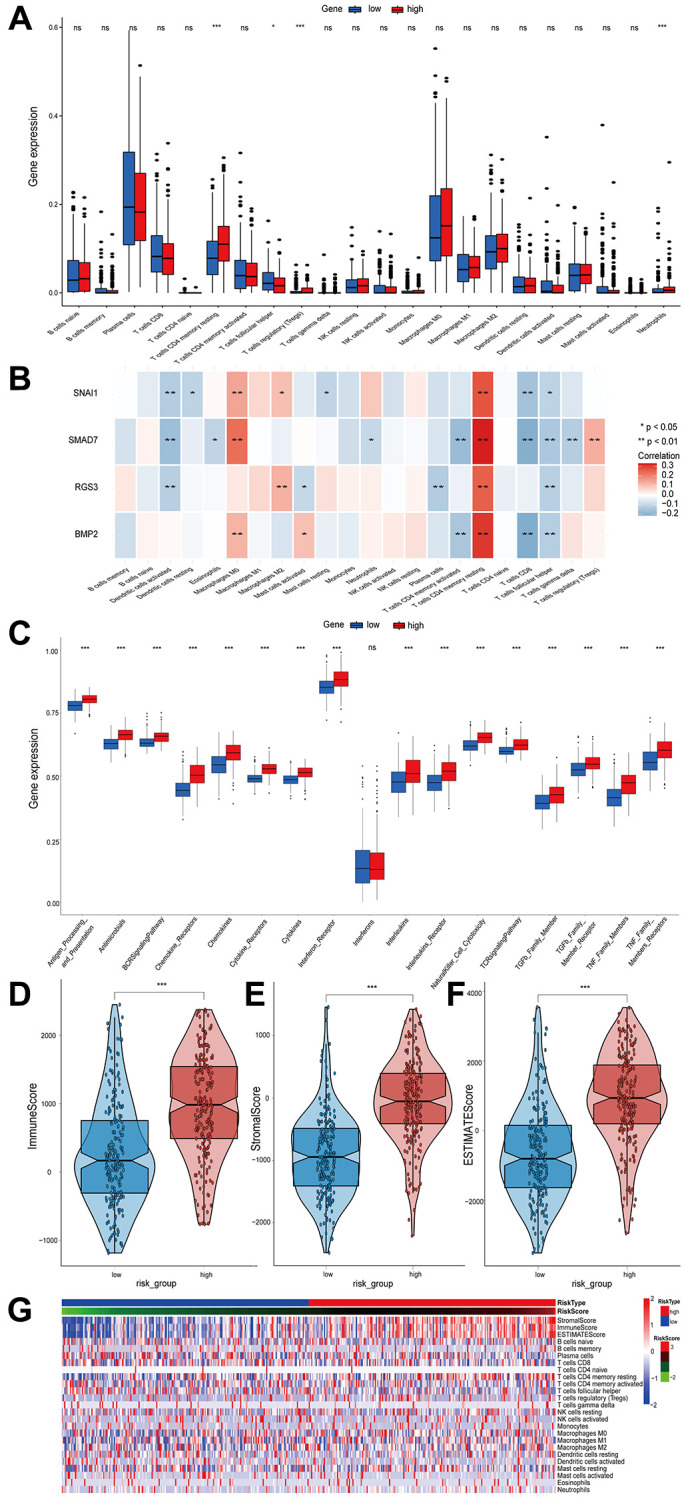
**The relationship between the infiltrated immune cells and risk score and the difference in immune score and immune pathways between high- and low-risk groups.** (**A**) Comparison of the infiltration level of 22 tumor-infiltrating immune cells between the high- and low-risk groups. (**B**) The correlation heatmap between 22 immune cells and EMT-RDGs. (**C**) Box plots showing the immune pathways analysis between high- and low-risk groups. (**D**–**F**) Boxplots of the immune score, stromal score, and ESTIMATE score. (**G**) Heatmap of immune cells and ESTIMATE score for high- and low-risk groups.

Based on the CIBERSORT algorithm, plasma cells accounted for the largest proportion, followed by macrophages M0 (Figure 5A). T cells CD4 memory resting, Tregs and neutrophils were significantly higher in high-risk patients, whereas the proportion of T cells follicular helper cells was higher in low-risk patients. The presence of immunosuppressive immune cells in the tumor microenvironment of the high-risk group was consistent with the poor prognosis of the high-risk group. The correlation between risk score and immune cell infiltration was analyzed in greater depth. The results demonstrated that the risk score was positively correlated with T cells CD4 memory resting, Tregs, and neutrophils, and negatively correlated with T cells follicular helper (Supplementary Figure 6). There was a strong correlation between the EMT-RDGs and most of the 22 immune cells used to construct the prognostic model (Figure 5B).

We investigated the immunological pathways given the existence of the large disparities in immune cell infiltration between high- and low-risk groups. The high-risk LUSC patients were significantly associated with pathways, including antigen processing and presentation, TGF-β signaling, and TCR signaling (Figure 5C). Previous studies have demonstrated that TGF-β is the key cytokine in the EMT process, which may partially account for the poor prognosis of the high-risk group. In addition, the ESTIMATE algorithm revealed that the high-risk group had a higher immune score (Figure 5D), stromal score (Figure 5E), and ESTIMATE score (Figure 5F), indicating that immune infiltration was higher in the high-risk group (Figure 5G).

**Figure 6:**
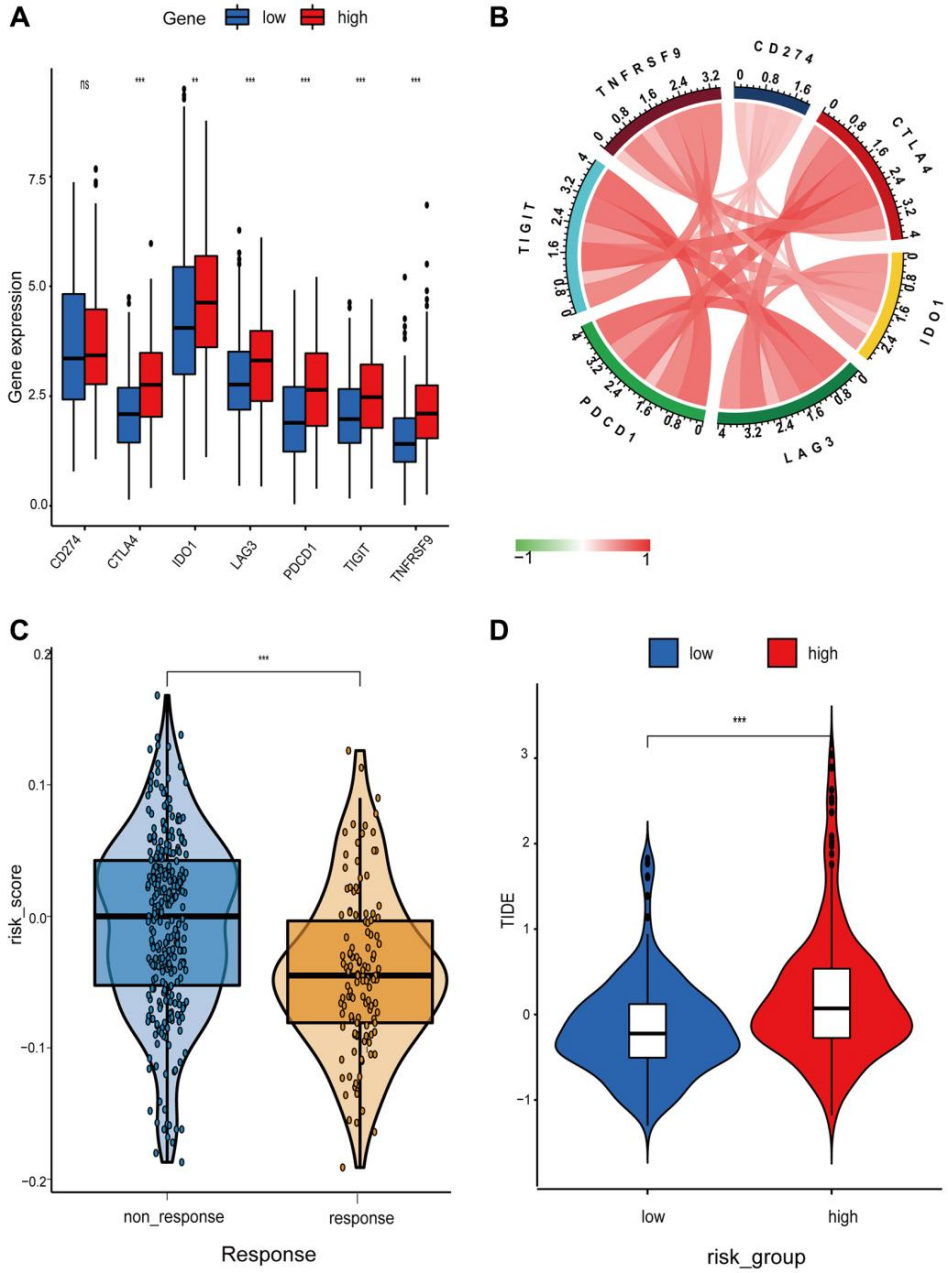
**Differential analysis of immune checkpoint and risk score between high- and low-risk groups.** (**A**) Boxplot showing differential expression of common immune checkpoint between high- and low-risk groups. (**B**) Chord diagram illustrating the relationship between the immune checkpoints. (**C** and **D**) Comparison of the immunotherapy response between high- and low-risk groups.

In recent years, anti-tumor immunotherapy for lung cancer has generated a great deal of interest. Seven immune checkpoint expressions were compared between high- and low-risk patients (Figure 6A and 6B). In the high-risk group, the expression of *CD274*, *CTLA4*, *IDO1*, *LAG3*, *PDCD1*, *TIGIT*, and *TNFRSF9* was elevated. The poor prognosis of the high-risk group may be attributable to the inhibition of the immune system by the high level of immune checkpoints expression. The TIDE algorithm and transcriptome data were then used to determine the correlation between immune infiltration and immunotherapy response. Results demonstrated that the high-risk group had a significantly higher probability of immunotherapy responders (Figure 6C and 6D).

TIDE predicted a poor immunotherapy response, even though the high-risk groups exhibited significantly stronger immune infiltration and immune checkpoint expression. Considering natural anti-tumoral system-mediated cytosolic immune response, we quantified the average expression levels of the granzyme A (GZMA) and perforin (PRF1) genes to evaluate the cytolytic activity (CYT). The results appeared consistent with the immune score, indicating that the high-risk group had significantly higher cytolytic and T cell inflammation scores (Supplementary Figure 7A and 7B). These results indicated that the tumors in the high-risk group were more immunogenic. It was suggested that the immune microenvironment, cytolytic activity, and T cell inflammation affected the prognosis of LUSC patients. The mRNAsi was a novel stemness index that was used to evaluate the dedifferentiation potential of tumor cells. In our study, the low-risk group had a higher mRNAsi score (Supplementary Figure 7C). It revealed that the low-risk group had tumor cells with a higher dedifferentiation potential. This may also partially explain the higher immunotherapy responses in the low-risk group.

### The responses of the high- and low-risk groups to drug treatments

**Figure 7:**
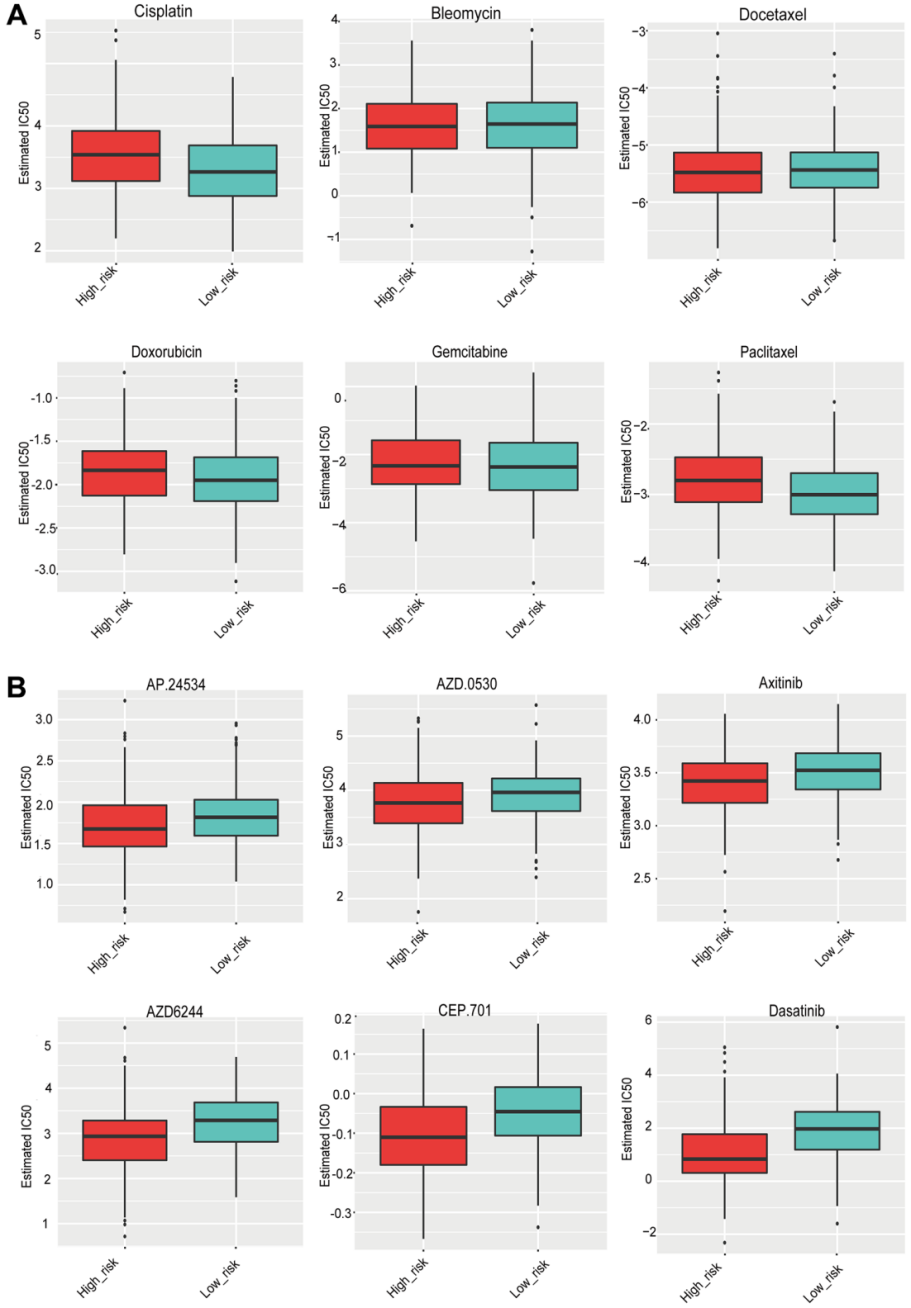
(**A**) The sensitivity of the high- and low-risk groups to six commonly used chemotherapeutic drugs. (**B**) The sensitivity of the high- and low-risk groups to targeted drugs with significant differences.

Based on the pRRophetic algorithm, 35 drugs exhibited statistically significant differences (Supplementary Table 1). The data was visualized using boxplots. The IC50 values for six common chemotherapeutic drugs (cisplatin, bleomycin, docetaxel, doxorubicin, gemcitabine, and paclitaxel) did not change significantly between the high- and low-risk groups (Figure 7A). Several drugs with significant differences were targeted drugs, including ponatinib, Saracatinib, Axitinib, and Lestaurtinib, all of which had a higher IC50 in the low-risk group (Figure 7B). In other words, patients in the high-risk group appeared to be more susceptible to these targeted drugs.

### Somatic mutation features in the high- and low-risk groups based on EMT-RDGs

We identified the mutation information in both the high- and low-risk groups. Among 418 LUSC patients from the TCGA database, 410 patients (98.09%) with single-nucleotide variant (SNV) data were selected for inclusion, with 208 patients in the high-risk group and the remaining 202 patients in the low-risk group. The waterfall was used to display the mutant situations of the top 20 genes with the highest mutation frequency. Missense mutations had the highest frequency of mutations, followed by nonsense mutations (Supplementary Figure 8A), and the number of single nucleotide polymorphism (SNP) was significantly larger than that of insertion (INS) or deletion (DEL). The most frequent nucleotide variation in the high-risk group was C > T, whereas in the low-risk group it was C > A (Supplementary Figure 8B–8D). The number of variants in each sample and different mutation types are indicated by different colors in Supplementary Figure 8E and 8F. The top ten mutant genes with the highest mutation frequency were displayed in Histograms (Supplementary Figure 8G). *TTN*, *TP53*, *MUC16*, *CSMD3*, *RYR2*, *LRP1B*, *USH2A*, *ZFHX4*, *SYNE1*, and *SPTA1* were the top 10 genes with the most frequent mutations in the high-risk group. *TTN*, *TP53*, *CSMD3*, *MUC16*, *SYNE1*, *RYR2*, *LRP1B*, *ZFHX4*, *USH2A*, and *FAM135B* were the top 10 genes with the most frequent mutations in the low-risk group. Based on the somatic mutational profiles of 410 LUSC patients, the MutSigCV algorithm identified 536 significantly mutated genes (SMGs) (*q* < 0.05). The top 10 most statistically significant driver genes were, *TP53*, *TTN*, *CSMD3*, *ZFHX4*, *FAM135B*, *CDH10*, *HCN11*, *ZNF804A*, *CDKN2A*, *and NFE2L* (Supplementary Figure 9). There were certain overlaps with genes with higher mutational frequency in high- and low-risk groups. *TP53* was identified as a cancer gene, with a high mutation tendency in both high- and low-risk groups. This may correlate with the prognosis of LUSC patients.

### Validation of the expression of four EMT-RDGs in LUSC

**Figure 8:**
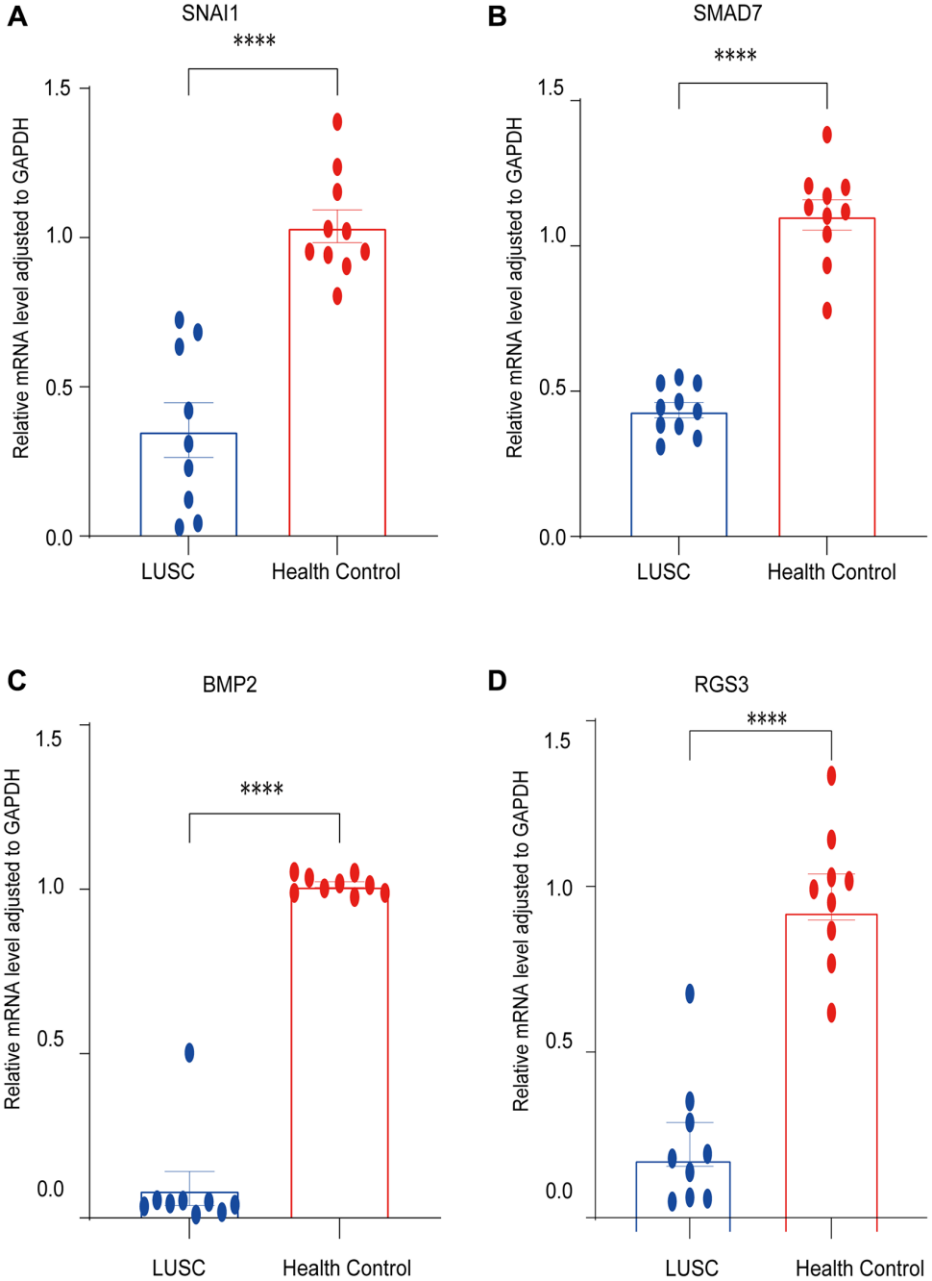
**Validation of the expression of EMT-RDGs by RT-PCR.** (**A**) *SNAI1*. (**B**) *SMAD7*. (**C**) *BMP2*. (**D**) *RGS3*.

qRT-PCR was used to determine the expression of four mRNAs in tissue extraction samples. The results showed that SNAI, SMAD7, BMP2, and RGS3 were significantly down-regulated, which was consistent with the bioinformatics analysis (Figure 8).

## DISCUSSION

Metastasis is an important contributing to poor prognosis in patients with LUSC [9]. The EMT process is core among the mechanisms driving tumor cell metastasis and chemoresistance [20, 21]. Recent studies have constructed EMT-RDGs risk score model to predict the prognosis of many types of cancer, such as colorectal cancer, liver cancer, and pancreatic ductal adenocarcinoma [22–29]. However, no study has tested whether ERGs-based models can predict the prognosis of LUSC patients. In this study, we systematically analyzed the expression profiles of ERGs in LUSC tissues in TCGA database. Risk score models were constructed based on EMT-RDGs for evaluating the prognosis of LUSC in clinical practice. The performance of the developed models was validated in GEO database. We also analyzed the mutational landscape, tumor immune environment, immune treatment, and drug sensitivity between high- and low-risk groups.

To construct the prognostic model, four EMT-RDGs (SNAI1, SMAD7, BMP2, and RGS3) were screened. SNAI1 is a zinc finger transcription repressor of E-cadherin that plays a role during early embryonic development and cell migration stages. It has been reported to repressed E-cadherin to influence EMT events [30]. It is, therefore, an important regulator of metastasis in lung cancer.

SMAD7 is an inhibitory Smad shown to inhibit TGF-β1 signaling through multiple mechanisms. Studies have indicated that TGF-β1 regulates EMT at the transcriptional and post-transcriptional levels, and that TGF-β1-induced EMT participates in lung cancer metastasis [31]. Thus, we speculate that the suppression of SMAD7 expression resulted in activation of the TGF-β1 signaling pathway leading to enhancement of metastasis in lung cancer.

Bone metastasis is one of the most common complications of advanced Non-Small Cell Lung Cancer Treatment (NSCLC). Data shows that activation of BMP2 signaling aggravates bone metastasis of NSCLC. In a previous study, BMP2 suppressed the protein expression of E-cadherin suggesting that BMP2 signaling regulates the morphological changes of cells induced by EMT [32]. It has been shown that EMT process increases the motility and invasion ability of cells [33]. In this study, we found that BMP2 expression was downregulated in the high-risk group. The mechanism involved need to be clarified in future studies.

RGS3 is a well-known regulator of G protein signaling pathways. For instance, it can inhibit the TGF-β/ SMAD signaling pathway in adventitial fibroblasts. Previously, it was reported that overexpression of microRNA-25 influenced the expression of RGS3 leading to the inhibition of apoptosis of lung cancer cells [34]. Moreover, microRNA-25 was found to be significantly upregulated in NSCLC tissues and negatively correlated the expression of microRNA-25 and RGS3 protein.

Based on the results provided above, the four cancer-related genes may have prognostic value in LUSC. However, further investigations are needed to verify this hypothesis and explore the underlying mechanisms.

A recent study showed that the EMT process influences the immune cellular infiltration status of tumor cells and cancer metastasis [35]. Against this background, we explored the immune microenvironment of high-risk and low-risk groups in this study. Results revealed a significant difference in immune infiltration of various cell types between the high- and low-risk groups. Specifically, patients in the low-risk group had higher abundance of follicular helper T cells, whereas those in the high-risk group had higher abundance of resting memory CD4 T cells, Tregs, and neutrophils. Evidence from previous studies indicate that tumor-infiltrating lymphocytes (TILs) are associated with the progression of various cancers [36]. For instance, infiltration level of cytotoxic T-cells, memory T cells, and helper T-cells was associated with a favorable prognosis [37]. Tumor immune-escape mechanisms have been a major limitation to the efficacy of drugs for controlling tumor progression [38]. Several mechanisms of tumor-immune escape have been reported including high number of immunosuppressive cells and overexpression of immune checkpoint molecules in tumor microenvironment [39]. The cancer immunoediting hypothesis states that during tumor development in immune-competent hosts, tumor cells with less immunogenicity are selected to escape antitumor activity [38]. Lower expression of mRNAsi in the high-risk group resulted in low immunogenicity. Therefore, the poor prognosis of patients in the high-risk group may be due to the strong immunosuppression and low immune activity in the tumor microenvironment.

Studies have demonstrated that immune checkpoint inhibitors are effective in patients with refractory malignancies including lung cancer. Therefore, immunotherapy is an emerging treatment for cancer. However, it is not effective in high-risk patients with high TIDE scores. This is attributed to the high tumor-infiltration of Tregs and stromal cells [40].

Compared to the low-risk group, although the high-risk group had higher immune cell infiltration and immune checkpoint expression, it had a higher TIDE score, and responded poorly to immunotherapy, which may be responsible for the high infiltration of stromal cells and Tregs in the high-risk group. High infiltration of stromal cells may lead to the formation of a barrier that prevents T cells from killing tumors [40], which inhibits immunotherapy response. In this study, a lower risk score was significantly associated with high expression of PD-L1 and TP53 mutation [41–43]. Patients in the low-risk group showed a high stemness index which had less differentiated [44]. These results demonstrate that patients with lower risk scores may benefit from immunotherapy. In the study by Denggang et al., it was found that the high-risk group with high expression of CTLA4 and TIM-3 had a poor response to immunotherapy [45]. Elsewhere, enrichment of stromal cells was revealed to be a cause of the poor response to immunotherapy in the high-risk group with elevated levels of immune-related gene pair (IRGP) [46]. Thus, while exploring the personalized immunotherapy and precise treatments, we cannot consider the effect of immune cells during treatment in isolation.

The bottlenecks in the treatment of LUSC made patients have to revert to traditional chemotherapy to improve prognosis. In our study, patients in the high-risk group showed better response to targeted drugs, such as Ponatinib, Saracatinib, and Axitinib. These drugs are often used to control NSCLC [47] and have been reported to confer good benefits in clinical trials [48, 49]. However, further clinical studies are required to investigate the clinical effects of these drugs. The above results suggest that the combination of traditional chemotherapy drugs and targeted drugs may be more therapeutically beneficial for high-risk groups. In conclusion, the data presented here indicate that the high-risk group may show good sensitivity to targeted drugs therapy, whereas the low-risk group may show good sensitivity to immunotherapy. Adoption of this criterion may reduce unnecessary treatments, decrease the economic burden on patients, and improve individualized treatment for patients.

Although the validation was performed in this study and the results obtained have considerable clinical relevance, there are some limitations to this work. Firstly, it was carried out based on the TCGA database, which lacked specific data on surgery, chemotherapy, and tumor size. Besides, some patients have undergone immune or targeted therapy, which may impact the prognosis analysis. Secondly, the number of samples in this study was relatively small. Therefore, future studies with a larger sample size are needed to further validate the performance of the signature. Thirdly, subgroup analyses were not performed due to the small sample size. For instance, the tumor stage was not different between subgroups, this may have been due to the different proportions of samples in different stages from the training and validation sets. In future, *in vitro* and *in vivo* experiments should be conducted to elucidate the biological functions of the four EMT-RDGs in LUSC.

## CONCLUSION

In this study, the expression pattern of LUSC patients was explored and a risk score model was constructed. In addition, the association of model with the prognosis, immune infiltration, and drug sensitivity of patients. The constructed model is expected to promote application of individualized therapies in LUSC patients.

## Supplementary Materials

Supplementary Figures

Supplementary Table 1
