# Supplementary material for: Comprehensive analysis of the role of a four-gene signature based on EMT in the prognosis, immunity, and treatment of lung squamous cell carcinoma
Source: Aging (Albany NY). 2023 Jul 17;15(14):6865–93. doi: 10.18632/aging.204878 (PMC10415548; doi:10.18632/aging.204878)
Supplement: Supplementary Figures [file aging-15-204878-s001.pdf]

SUPPLEMENTARY FIGURES

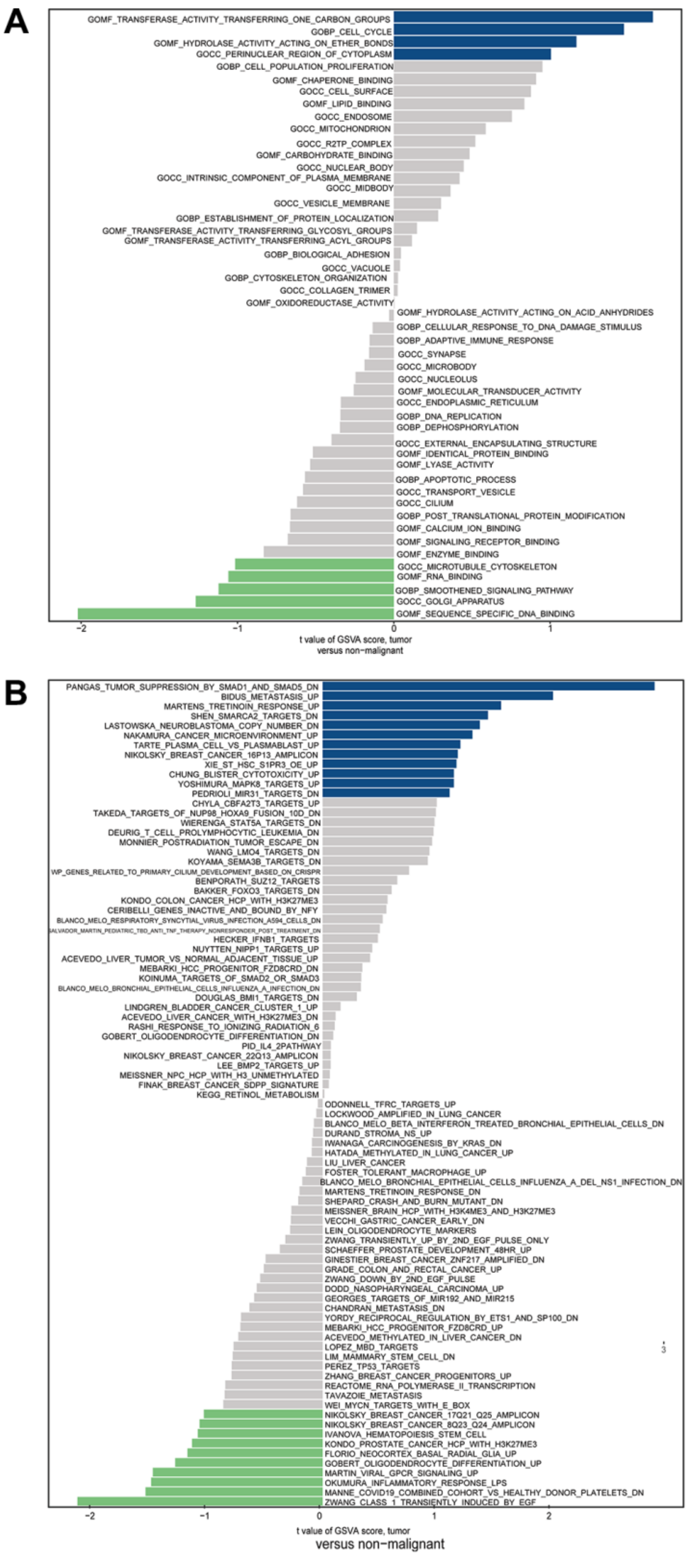

Supplementary Figure 1. A barplot depicting the GO enrichment (**A**) analysis and GSVA analysis of KEGG (**B**). The blue bands indicate a positive correlation, whereas the green bands indicate a negative correlation.

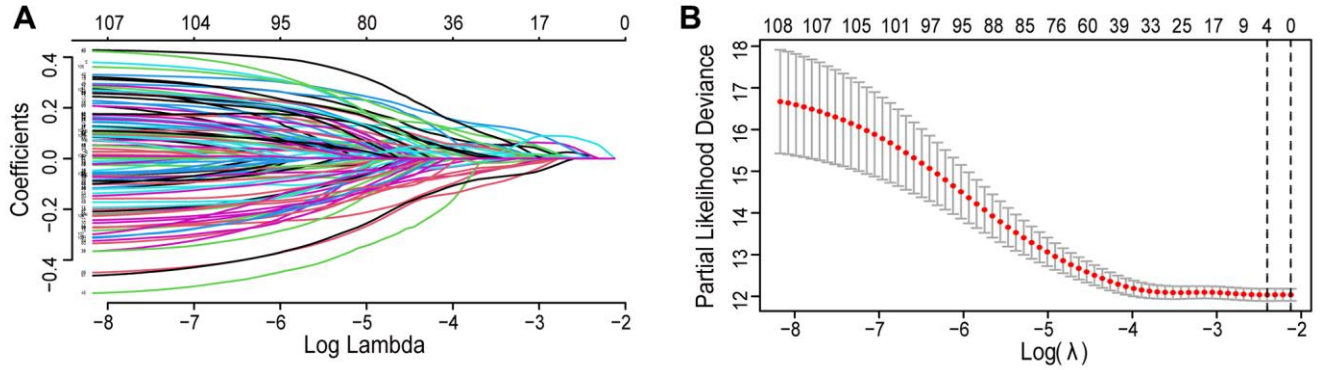

Supplementary Figure 2. Establishment of a prognostic model using LASSO regression analysis. (**A**) LASSO coefficient profiles for 108 EMT-RDGs. Each curve corresponds to a single gene. (**B**) Optimal parameter (lambda) selection in the LASSO model.

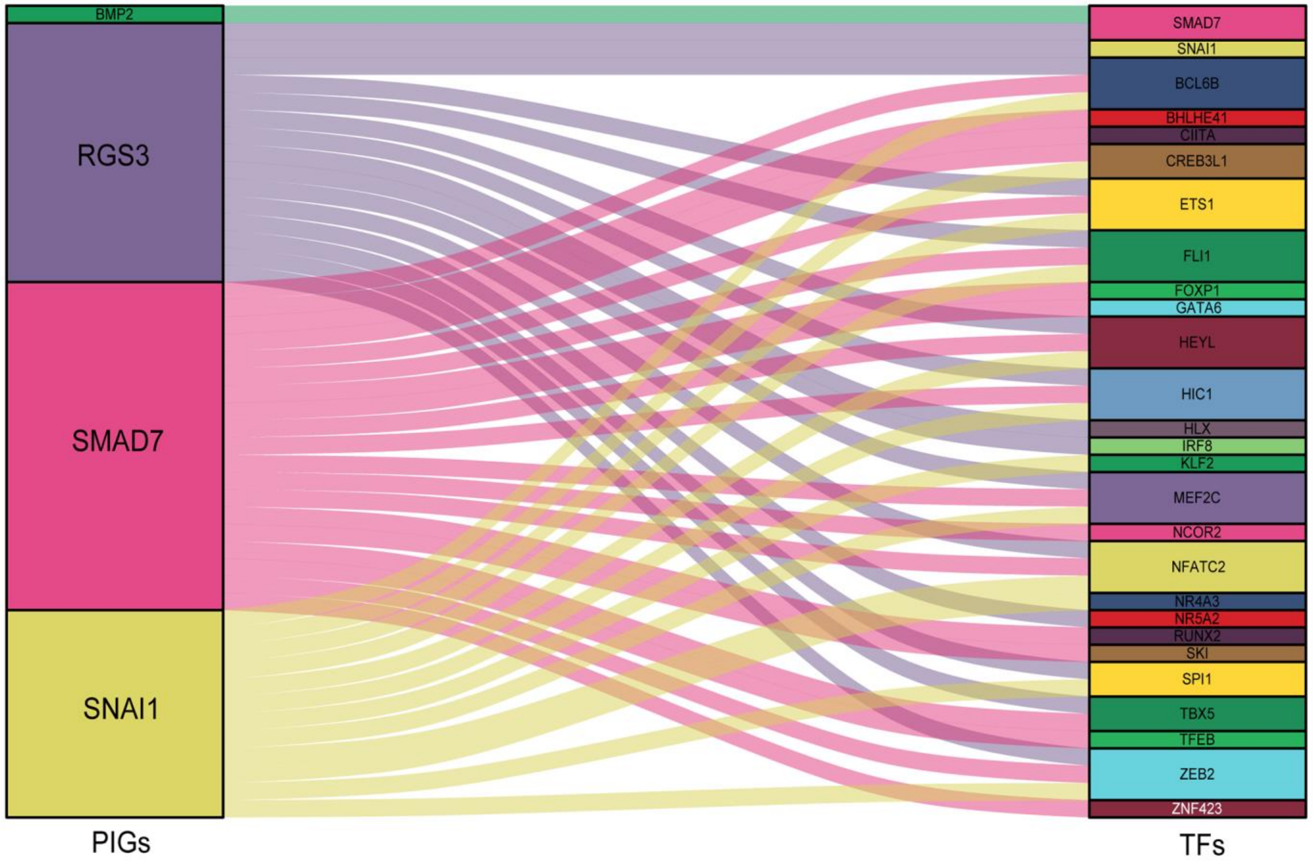

Supplementary Figure 3. Sankey diagram. The correlation between TFs and EMT-RDGs.

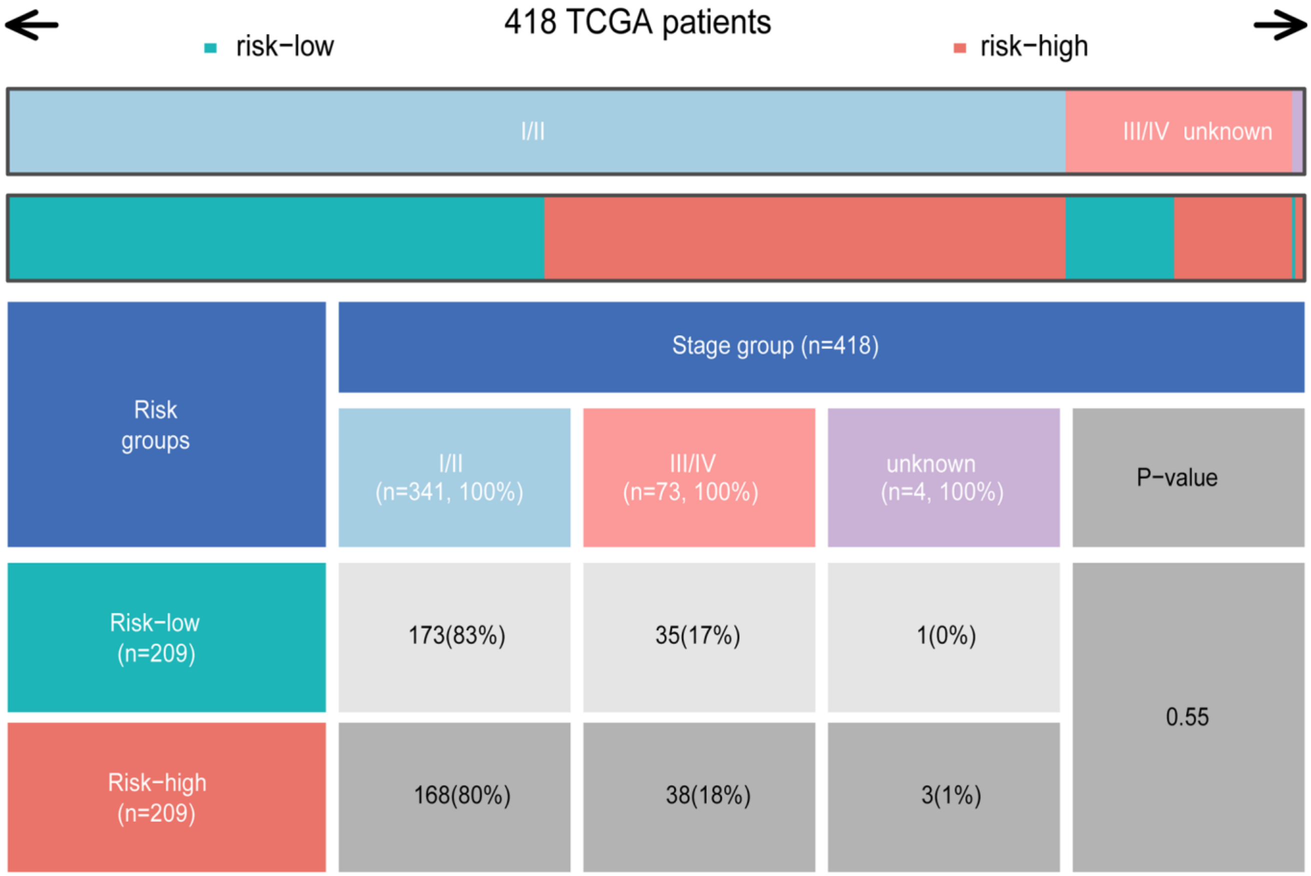

Supplementary Figure 4. The clinicopathological characteristics of the high- and low-risk groups.

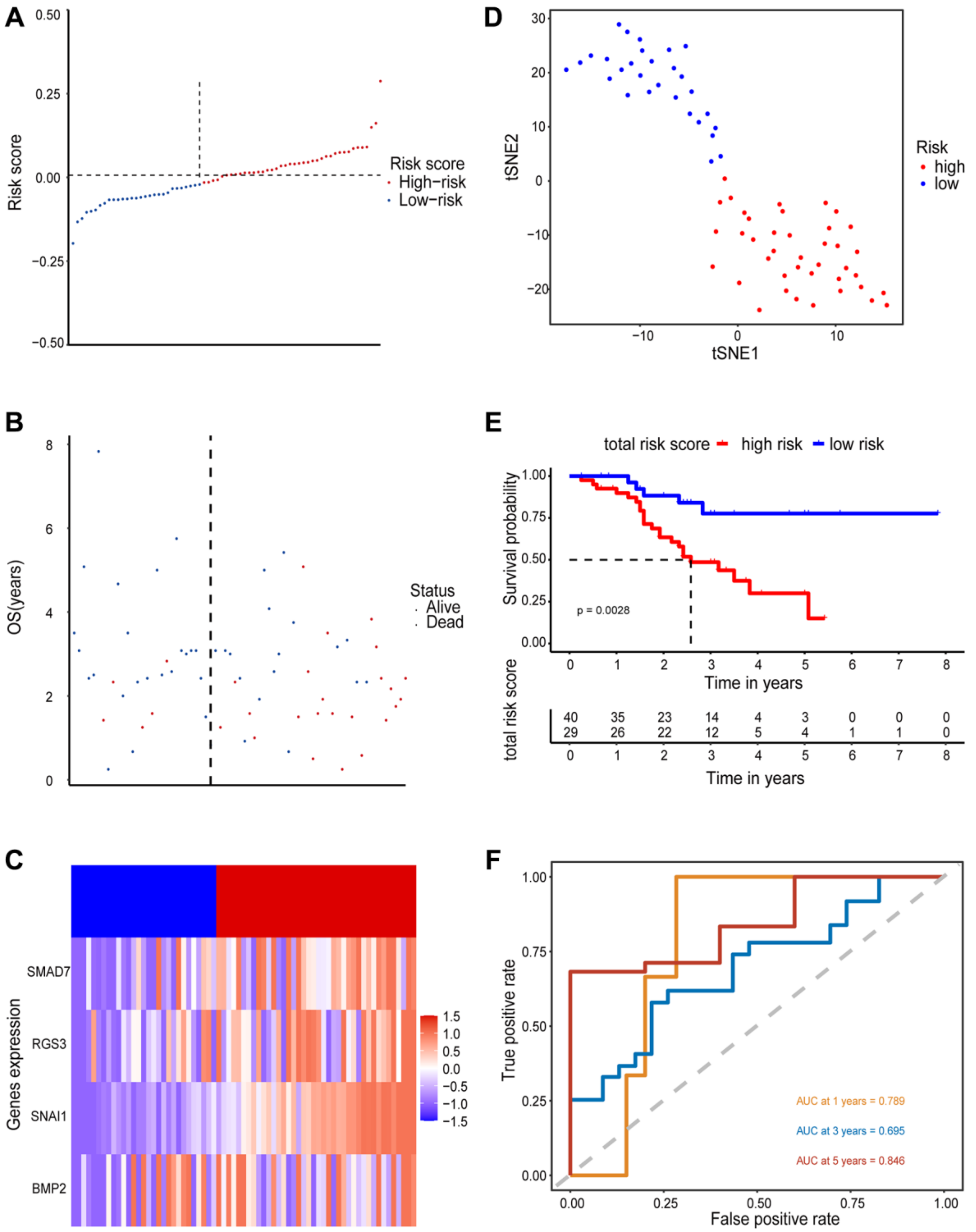

Supplementary Figure 5. Prognostic value of 4 EMT-RDGs in the validation set. (**A**) A risk curve based on the risk score of each sample. (**B**) A scatter plot showing the survival status of each sample. (**C**) A heatmap of 4 EMT-RDGs. (**D**) t-SN plot of high-and low-risk groups based on the four-gene prognostic model. (**E**) Kaplan-Meier survival curve analysis. (**F**) ROC curves for the 1, 3, and 5-year survival prediction.

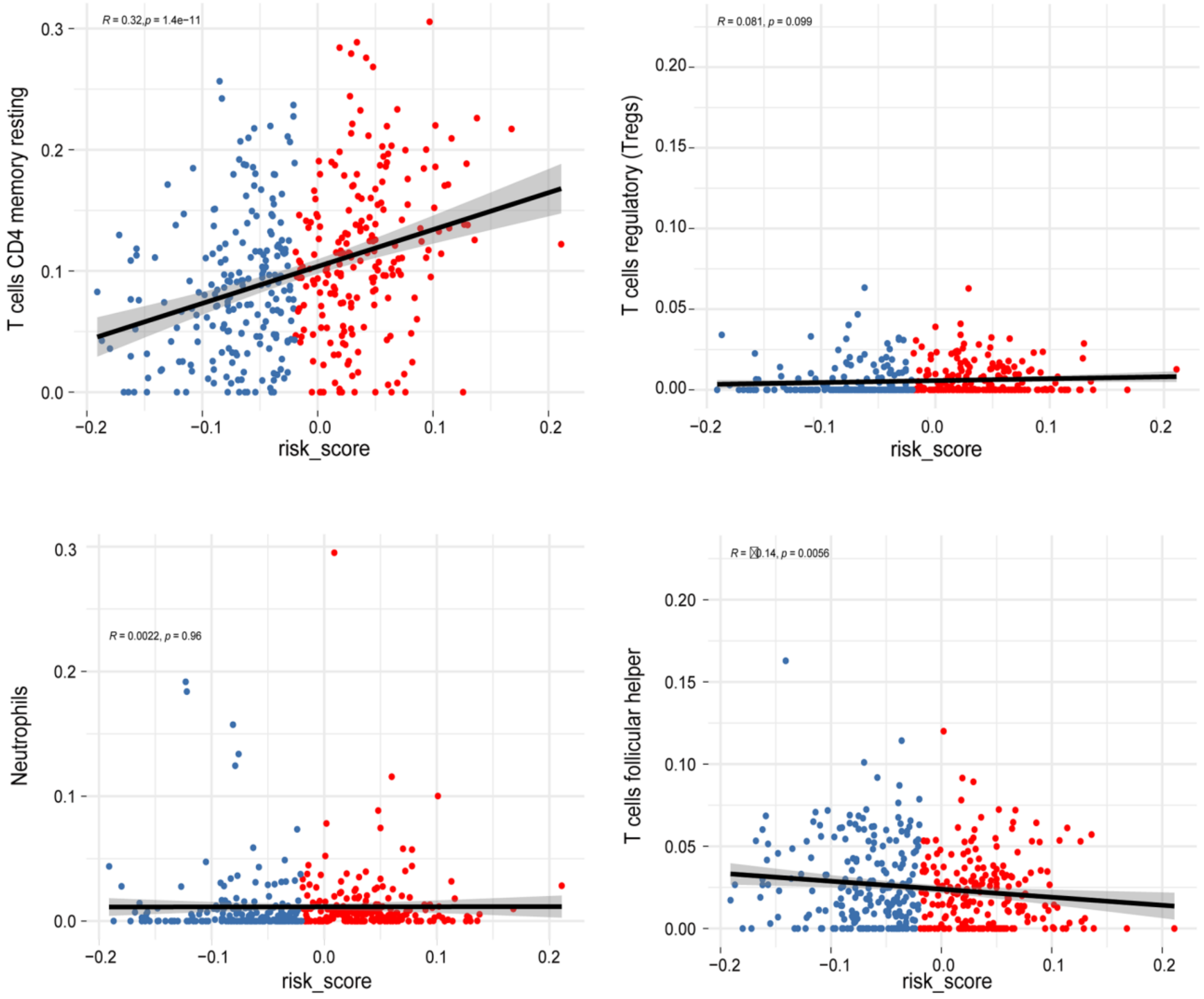

Supplementary Figure 6. The statistically significant relationships between risk score and level of immune cell infiltration.

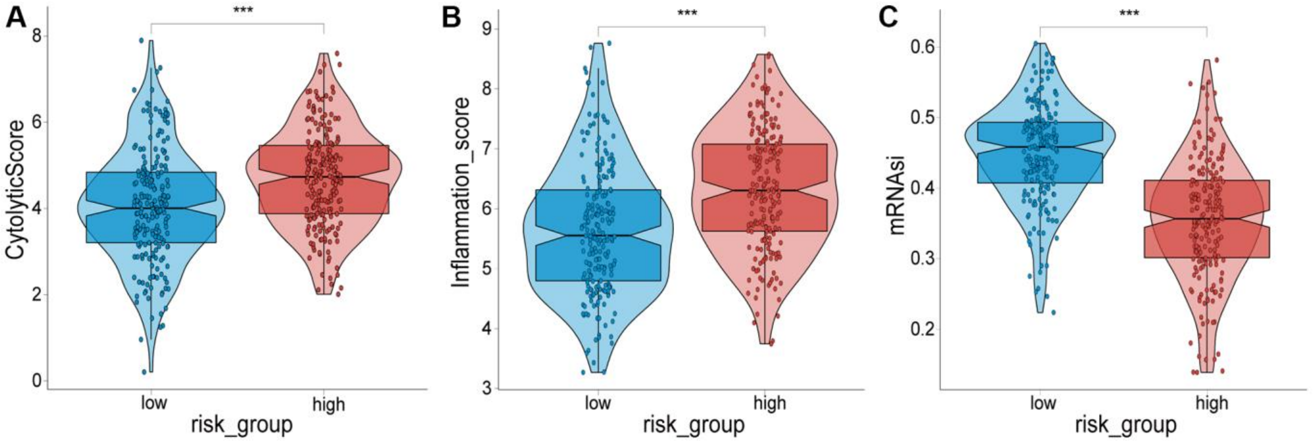

Supplementary Figure 7. Differential analysis of cytolytic activity scores, T cell inflammation scores, and mRNAsi indexes between the high- and low-risk groups. (**A**) Difference in cytolytic activity scores between the high- and low-risk qroups. (**B**) Differences in T cell inflammation scores between the high- and low-risk groups. (**C**) Difference in mRNAsi indexes between the high- and low-risk groups.

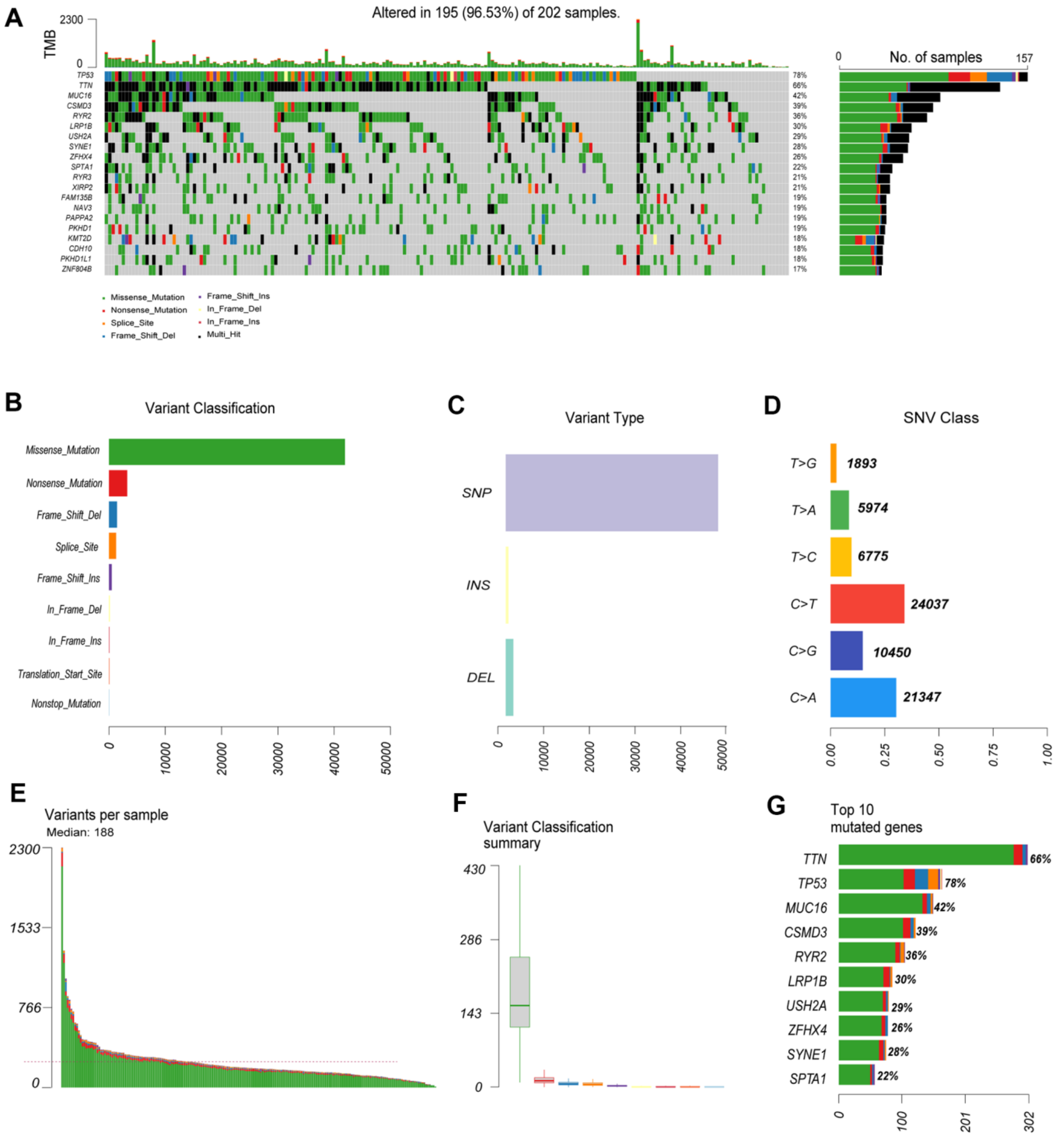

Supplementary Figure 8. Information on somatic mutations in LUSC patients in the high-risk group. (**A**) The waterfall plot shows mutation information for each gene in LUSC patients. Rectangles with different colors represent different mutation types. (**B**–**D**) Classification of different mutation types. The most common type of mutation in the summarized figure was a missense mutation. (**E**) The number of variants in each sample. (**F**) Box plot. Different colors represent different mutation types. (**G**) Histograms of the top ten most frequently mutated genes in LUSC.

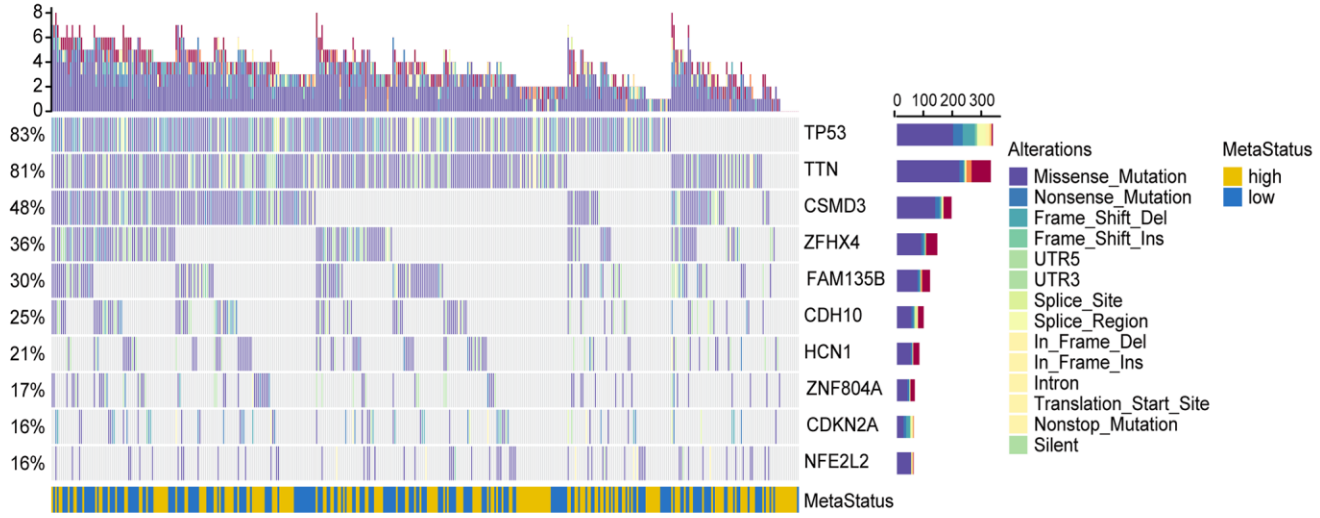

Supplementary Figure 9. The mutational landscape of SMGs in LUSC patients. Top: The number of aberrations detected within each sample is shown. Middle: Significantly mutated genes are colored based on the mutation types. Left: The mutation frequency of each gene. Right: The top ten significantly mutated genes, ranked by *q*-value according to MutSigCV analysis and the number of mutations per gene. Bottom: High- and low-risk groups.
