# Supplementary material for: Comprehensive analysis of the role of a four-gene signature based on EMT in the prognosis, immunity, and treatment of lung squamous cell carcinoma
Source: Aging (Albany NY). 2023 Jul 17;15(14):6865–93. doi: 10.18632/aging.204878 (PMC10415548; doi:10.18632/aging.204878)
Supplement: Supplementary Table 1 [file aging-15-204878-s002.pdf]

SUPPLEMENTARY TABLE

Supplementary Table 1. The IC50 of 65 chemotherapy drugs in the high- and low-risk groups of LUSC patients.

Drug's name	*P*.value
A.443654	1.00000000
BIBW2992	1.00000000
BI.2536	1.00000000
BIRB.0796	1.00000000
CCT018159	1.00000000
Gefitinib	1.00000000
Erlotinib	0.99999999
Bosutinib	0.99999983
Cisplatin	0.99998044
Epothilone.B	0.99992999
ATRA	0.99791900
Etoposide	0.99735393
FH535	0.99688335
Doxorubicin	0.95712317
BI.D1870	0.91972925
Elesclomol	0.87438764
EHT.1864	0.71950857
Gemcitabine	0.64221140
Cyclopamine	0.63948724
CGP.082996	0.59314005
ABT.888	0.38657311
AMG.706	0.31067710
ABT.263	0.27994608
Docetaxel	0.22039561
AKT.inhibitor.VIII	0.18308981
BAY.61.3606	0.14699632
Bleomycin	0.13859567
Bexarotene	0.09412018
Camptothecin	0.08357952
AICAR	0.05181055
Bortezomib	0.00748255
CCT007093	0.00508396
GNF.2	0.00198718
AP.24534	0.00186805
CMK	0.00053376
AZD.0530	0.00022121
Cytarabine	0.00018178
Axitinib	0.00009021

AG.014699	0.00008762
AUY922	0.00004240
FTI.277	0.00003666
AZD7762	0.00001216
BMS.708163	0.00000894
Embelin	0.00000837
AZD8055	0.00000038
AZD.2281	0.00000019
AZD6244	0.00000011
CHIR.99021	0.00000000
AZ628	0.00000000
CEP.701	0.00000000
A.770041	0.00000000
AS601245	0.00000000
DMOG	0.00000000
Bryostatin.1	0.00000000
Bicalutamide	0.00000000
CI.1040	0.00000000
BMS.509744	0.00000000
CGP.60474	0.00000000
BX.795	0.00000000
AZD6482	0.00000000
Dasatinib	0.00000000
BMS.536924	0.00000000
BMS.754807	0.00000000
GDC.0449	0.00000000
GDC0941	0.00000000
